# A REDCap-based model for electronic consent (eConsent): Moving toward a more personalized consent

**DOI:** 10.1017/cts.2020.30

**Published:** 2020-04-03

**Authors:** Colleen E. Lawrence, Leah Dunkel, Mark McEver, Tiffany Israel, Robert Taylor, Germán Chiriboga, Karin Valentine Goins, Elizabeth J. Rahn, Amy S. Mudano, Erik D. Roberson, Carol Chambless, Virginia G. Wadley, Maria I. Danila, Melissa A. Fischer, Yvonne Joosten, Kenneth G. Saag, Jeroan J. Allison, Stephenie C. Lemon, Paul A. Harris

**Affiliations:** 1 Vanderbilt Institute for Clinical and Translational Research (VICTR), Vanderbilt University Medical Center, Nashville, TN, USA; 2 Vanderbilt Institute for Medicine and Public Health, Vanderbilt University Medical Center, Nashville, TN, USA; 3 Vanderbilt Office of Research, Vanderbilt University Medical Center, Nashville, TN, USA; 4 Department of Medicine, University of Massachusetts Medical School, Worcester, MA, USA; 5 Department of Population and Quantitative Health Sciences, University of Massachusetts Medical School, Worcester, MA, USA; 6 Division of Clinical Immunology/Rheumatology, University of Alabama at Birmingham, Birmingham, AL, USA; 7 Department of Medicine, The University of Alabama at Birmingham School of Medicine, Birmingham, AL, USA; 8 Department of Neurology, Alzheimer’s Disease Center, The University of Alabama at Birmingham, Birmingham, AL, USA; 9 Department of Biomedical Informatics, Vanderbilt University Medical Center, Nashville, TN, USA

**Keywords:** Consent, REDCap, personalized, electronic, community engagement

## Abstract

**Introduction:**

The updated common rule, for human subjects research, requires that consents “begin with a ‘concise and focused’ presentation of the key information that will most likely help someone make a decision about whether to participate in a study” (Menikoff, Kaneshiro, Pritchard. *The New England Journal of Medicine*. 2017; **376**(7): 613–615.). We utilized a community-engaged technology development approach to inform feature options within the REDCap software platform centered around collection and storage of electronic consent (eConsent) to address issues of transparency, clinical trial efficiency, and regulatory compliance for informed consent (Harris, et al. *Journal of Biomedical Informatics* 2009; **42**(2): 377–381.). eConsent may also improve recruitment and retention in clinical research studies by addressing: (1) barriers for accessing rural populations by facilitating remote consent and (2) cultural and literacy barriers by including optional explanatory material (e.g., defining terms by hovering over them with the cursor) or the choice of displaying different videos/images based on participant’s race, ethnicity, or educational level (Phillippi, et al. *Journal of Obstetric, Gynecologic, & Neonatal Nursing*. 2018; **47**(4): 529–534.).

**Methods:**

We developed and pilot tested our eConsent framework to provide a personalized consent experience whereby users are guided through a consent document that utilizes avatars, contextual glossary information supplements, and videos, to facilitate communication of information.

**Results:**

The eConsent framework includes a portfolio of eight features, reviewed by community stakeholders, and tested at two academic medical centers.

**Conclusions:**

Early adoption and utilization of this eConsent framework have demonstrated acceptability. Next steps will emphasize testing efficacy of features to improve participant engagement with the consent process.

## Introduction

In 2015, the Clinical Trials Transformation Initiative (CITI) launched the Informed Consent Project to assess recommendations and observations from experts in the field of consent. The study collected data from 25 experts, representing an array of clinical trials, and concluded that the current informed consent document must be revised to be shorter, clearer, and more understandable [1]. One way to address these concerns is to move the consent form from a paper document to an electronic interface, thereby enabling the use of electronic media, such as videos, interactive comprehension questions, and avatars (i.e., virtual characters), to support a customized and engaging consenting interface that could substantially enhance a research volunteer’s ability to read, retain, and comprehend key information on the study and their role as a participant [2,3].

Electronic consent (eConsent) is defined by the US Food and Drug Administration (FDA) as “the use of electronic systems and processes that may employ multiple electronic media, including text, graphics, audio, video, podcasts, passive and interactive Web sites … to convey information related to the study and to obtain and document informed consent” [4]. With the possibility for using technology to convey complicated information, research teams are considering eConsent over static paper documents for improving the overall consenting experience (e.g., increased participant comprehension, participant appeal, efficiency, and bi-directional ease of use) [5–8]. The enhanced features within eConsent provide an opportunity to facilitate recruitment and retention of participants with particular opportunities to engage underrepresented minority groups through improving understanding and the consenting experience [9]. Another driver for eConsent is the movement toward more pragmatic trials that either (1) engage directly with patients using centralized resources for consenting or (2) utilize research sites that do not typically conduct clinical trials and where paper informed consent presents a barrier to participation. eConsent could make it easier for community sites to do clinical trials and thereby increase their generalizability. With the possibilities of eConsent to improve the participant experience and extend the reach of pragmatic clinical trials, there are new considerations around regulatory compliance, remote consenting (i.e., consenting online rather than in person), proper documentation of consent, and cyber security as it pertains to protected health information. The FDA has released guidance to address these considerations with recommendations for Institutional Review Boards (IRBs), investigators and sponsors on the use of eConsent and the processes associated with obtaining informed consent for clinical investigations using various forms of digital electronic data [10].

Our eConsent framework is constructed using REDCap (**R**esearch **E**lectronic **D**ata **Cap**ture), a secure, web-based platform for building and managing online surveys and databases. REDCap was developed at Vanderbilt in 2004 to provide research teams a means to collect and manage research study data [11]. REDCap allows research teams to design data management solutions for projects using case report forms and/or participant-facing surveys. REDCap is ideal for eConsent as it allows for sharing of data within and across institutions (i.e., for multicenter trials), requires user authentication and can assign data access rights based on user role, allows for field-level data validation, and has mechanisms for ensuring data quality and integrity via an audit trail feature [11]. REDCap permits document storage, central data storage, and backups [11]. The REDCap platform, codebase, and REDCap consortium support are provided at no cost by our team at Vanderbilt with any academic, nonprofit, or government agency using a simple licensing model [12]. As of March 2020, REDCap was supporting >1 million end users at >4000 licensed REDCap Consortium partners across 137 countries.

The REDCap eConsent framework was informed by nearly 5 years of discussion with researchers, Vanderbilt IRB analysts and legal counsel, developers, and prospective participant users, in addition to conversations with stakeholders across the Clinical and Translational Science Award (CTSA) Network and the Trial Innovation Network (TIN) [13,14]. These conversations, termed “community-engaged technology development,” began at project inception and continued through pilot testing and full deployment of the framework. The process informed the specifications for eConsent portfolio content in terms of regulations and key design elements (e.g., “wet signature” – digital capture of physical signature, reviewer mode, version control management, and the look and feel of avatars and videos).

Private vendors market eConsent software (e.g., Mytrus, DatStat); however, these packages are often costly and do not translate well to the complete range of needs for researchers and their participants [15,16]. We sought to develop a generalizable REDCap-based eConsent framework to meet the following specifications:

1. The framework must address all regulatory requirements for informed consent including 21 CFR Part 11 compliance, version control, facilitation of critical elements of consent, and be easy for IRBs, study teams, sponsors, and monitors to review.
2. The framework and methods must be agile to accommodate tracking of multiple versions of the same consent, amendments, and multiple consent types (e.g., paediatric, adult) within the same study.
3. The framework must be easily customizable to:
  - the needs of the participant – that is, adaptable to “meet the participant where they are” in terms of health literacy, interest, and knowledge.
  - the needs of a given study team, study type, or patient population.
4. The framework should allow for collection and tracking of metrics related to a participant’s interaction with the eConsent platform (i.e., time spent watching videos, total time interacting with the consent).
5. The framework should be scalable/transferrable and easy to adopt for REDCap Consortium partners at little or no cost.

Addressing personalization of eConsent involves tailoring to the needs of diverse prospective participants, including underrepresented minority groups. There are many recognized limitations of consent documents: length, complexity, lack of clear understanding as to what information is essential to include in a consent, inability to have one document that meets all the information and communication needs of prospective participants, and no good methods for evaluating the adequacy of informed consent from the participant perspective [17]. All of these deficits in the consent form itself make informed consent inaccessible to those with limited health literacy, defined as “the capacity to obtain, process, and understand basic information needed to make informed decisions about research participation,” which can preclude research participation [18–20]. Our eConsent framework was developed as part of an intervention package for the STRIDE project.

The STRIDE project is a collaboration with the University of Massachusetts Medical School (UMass), the University of Alabama at Birmingham (UAB), and Vanderbilt University Medical Center (VUMC) with the goal of developing, testing, and disseminating an integrated multilevel, health literacy targeted intervention to improve access for African Americans and Latinos in translational research. STRIDE interventions include the eConsent framework, storytelling videos, and simulation training [21,22]. Despite disparities in leading causes of death, morbidity, and disability, African Americans and Latinos are underrepresented in important research studies [23]. While not the only reason for decreased clinical trial participation, there are data that show the rate of low health literacy is higher among racial/ethnic minorities than non-Latino whites, thus providing a potential opportunity to increase minority participation in clinical trials by improving the understandability of the consent document [24,25].

Herein, we describe the development of our REDCap-based eConsent framework and associated features and illustrate how our eConsent process was deployed and tested for acceptability by community and research team stakeholders.

## Materials and Methods

### REDCap External Modules

REDCap external modules are built, deployed, and maintained by REDCap Consortium partners to extend REDCap beyond its standard distribution version. External module creators can disseminate their work for use by REDCap Consortium partners by publishing in the REDCap Repo (i.e., a centralized repository of curated External Modules that can be downloaded to local REDCap instances) [26]. As we designed the eConsent framework, our REDCap team decided that some features should be built into the main REDCap distribution codebase while other more experimental feature should be built using external modules.

### Soliciting Stakeholder Feedback to Build eConsent

#### Community engagement studios

The Community Engagement Studio (CES) program, developed at VUMC, is a “structured program that facilitates project-specific input from community and patient stakeholders to enhance research design, implementation, and dissemination [27]. This model was utilized to inform aspects of eConsent feature development.

#### Community Investigators

Community Investigators (CIs) are members of minority and underrepresented populations and brought diverse perspectives based on a wide range of education levels, health care, and life experiences. The CIs meet monthly as part of the STRIDE team.

### Pilot Testing eConsent

Pilot testing of eConsent was conducted by UAB in conjunction with the VUMC/REDCap teams. The teams sought to seamlessly integrate the evolving eConsent protocols into the workflow of an exemplar clinical trial without increasing burden on the research teams. User testing with research staff, prospective research participants, and Principal Investigators of the exemplar study was used to determine framework acceptability for ease of use, adequacy of the material covered, level of comfort with eConsent by both research staff and prospective research participants, overall satisfaction, perceived eConsent utility, barriers to implementation, and additional training needs.

### eConsent Portfolio

Based on the emphases of the STRIDE project and conversations with multiple research teams at VUMC and REDCap Consortium partners, we focused on enhancing two existing REDCap features and developing eight new features for inclusion in the REDCap eConsent portfolio. Existing features that were enhanced to support eConsent were Branching Logic – Comprehension Questions and PDF – Consent. New features are (1) video library, (2) wet signature, (3) avatar, (4) in-line descriptive popups, (5) analytics module, (6) PDF-consent document repository, (7) document vault, and (8) Part 11 Certification for the eConsent framework. All features in the eConsent portfolio are available to users at no cost except the avatar feature.

#### Branching logic – comprehension questions

Information is displayed sequentially to users and embedded program branching logic filters content such that participants are presented with questions or content derived from answers to previous questions. Branching logic can be used in eConsent to create comprehension questions that utilize logic to confirm participant responses, direct participants to the correct information within the document, or help study teams assess concepts which are confusing to study participants.

#### Feature 1: Video library

The video library presents complex procedures and research concepts that can be used to increase accessibility of study materials for persons of varying health literacy as part of the consent process. To develop the video library, we conducted a needs assessment by surveying publicly available YouTube channels associated with >60 CTSA institutions to review available educational videos. The assessment was designed to help community stakeholders provide input on desired video content and key video features needed to support eConsent. Input from CIs affiliated with the STRIDE team and community stakeholders was used to inform all aspects of video development including imagery, lighting, voiceovers, background music, representativeness of characters, and the interactions depicted between characters. Community members contributed to the videos by providing voiceovers and serving as actors. Using feedback from the needs assessment and community feedback, we developed initial content for the video library. Draft video scripts were reviewed by the VUMC IRB and Effective Health Communication Core to assess appropriateness and readability. Filming was done at VUMC. ARTMAGIC LABS (Nashville, TN) provided filming, editing, and closed captioning services. Draft videos were reviewed by community members at UAB, UMass, and VUMC. Videos are publicly available (https://www.youtube.com/channel/UCKOqWFdtVU7XsxWs2fpfvNQ) and can be easily inserted into REDCap eConsents.

#### Feature 2: Wet signature

We developed a field type for capturing wet signatures. REDCap allows for various field types which specify how information is displayed on the data collection instrument (i.e., how it will appear to the participant). The signature field allows users to sign a document with a mouse, stylus, or their finger. The signature is captured, stored, and appended to the eConsent as a PNG image file with a timestamp and rendered in the signed PDF documentation used to record eConsent transactions.

#### Feature 3: Avatar

**Fig. 1.**
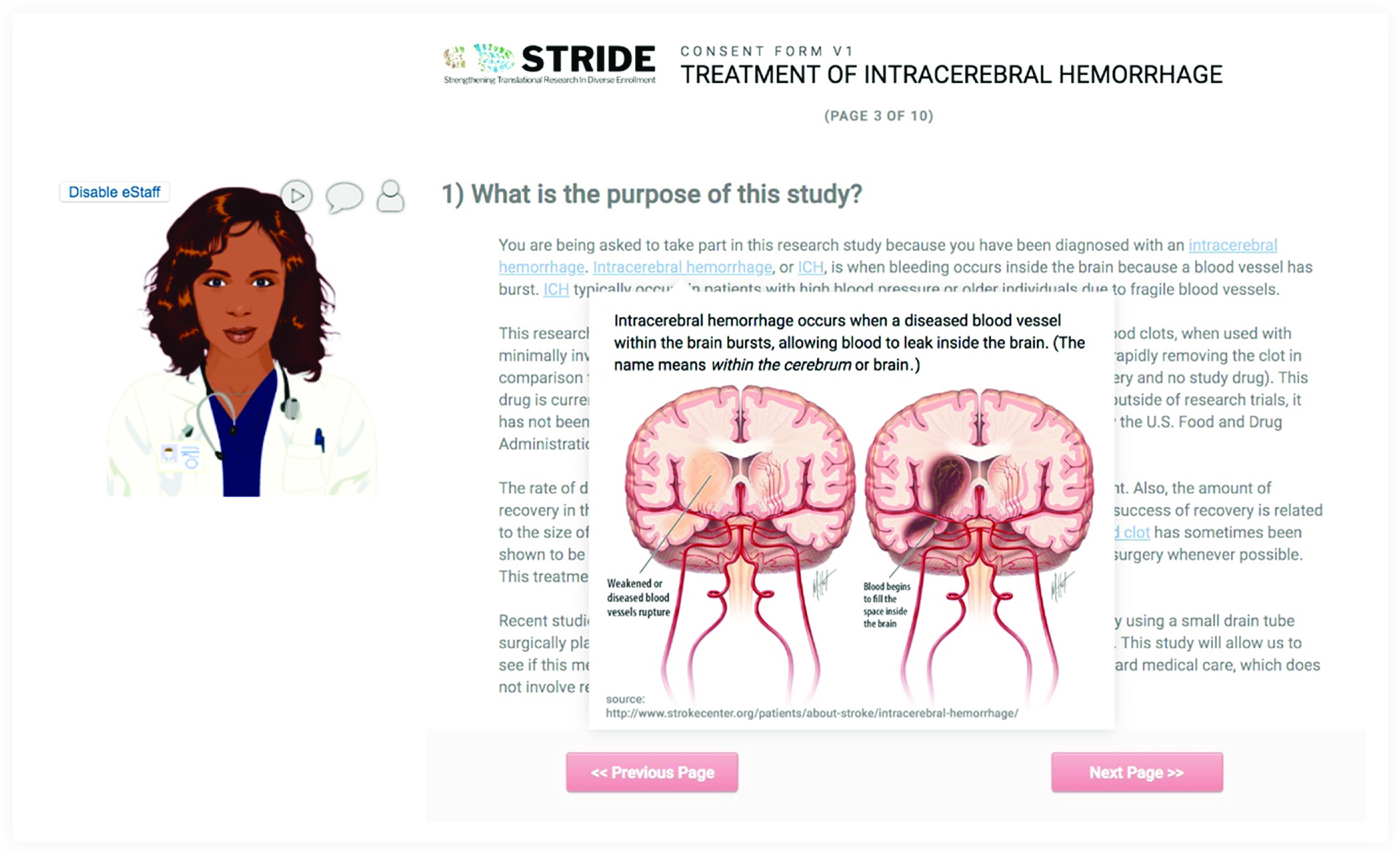
The REDCap-based model of eConsent allows for the incorporation of avatars, virtual assistants that can be self-selected by participants. Additional features include the use of “hover and click” in-line descriptive popups that allow participants the option to obtain more information (e.g., pronunciation, images, and definitions) about key words in the consent.

The avatar feature provides a virtual assistant (eStaff) to guide a participant through the eConsent with voiceover instruction or clarification. We used Oddcast media software studio to create scripted avatar messaging for use with eConsent [28]. Although avatar functionality is not native in the REDCap software platform, we used REDCap’s External Module architecture to build the support infrastructure. This external module can be shared but requires adopting institutions to license and support Oddcast media software. When deployed for a project, avatars can be customized in appearance, voice, and scripts (Fig. 1). Using the avatar module requires secure, real-time “text to speech” information exchange with the Oddcast 3rd-party solution provider. To ensure data privacy, all information is encrypted during transmission, and we recommend omission of all participant identifiers in avatar-based communication scripts.

#### Feature 4: In-line descriptive popup

This external module allows researchers to deploy supplemental information (e.g., text definitions, pronunciations, explanatory images) for review by prospective research participants. Supplemental information is linked to individual words or phrases and highlighted within an eConsent document so that when a prospective participant hovers over a linked word, supplemental information is displayed in a popup window. This feature allows prospective participants to decide how much detail they want from the eConsent document (Fig. 1).

#### Feature 5: Analytics module

This external module functions in the background of eConsent survey mode to capture metrics about how a participant interacts with features of the survey, such as videos, avatars, and in-line descriptive popups.

### eConsent Transaction and PDF Rendering

#### PDF-consent

When a REDCap survey or eConsent is completed, participants can have a PDF copy of their consent printed or emailed to them. The “PDF-consent” tool generates a streamlined PDF that removes unselected choices and irrelevant branching logic content (e.g., material not shown to participant because it was not relevant).

#### Feature 6: PDF-consent document repository (i.e., “auto-archiver”)

The “Auto-Archiver + eConsent framework” option adds two things to the typical REDCap survey process. (1) Before a participant completes the survey, a certification page is added to the end of the survey that displays an in-line PDF copy of their responses in which they are asked to confirm that all information in the document is correct. The consent documentation will not be considered complete until the participant fulfills the certification step. (2) Upon completion of the eConsent, a static copy of their responses in the form of a consent-specific PDF will be stored in the project’s File Repository.

#### Feature 7: Document vault

We established within the REDCap eConsent framework module the ability for institutions to specify settings for a local secure file transfer protocol (sFTP) site. The rationale behind this decision was (1) to allow redundant storage outside the REDCap system and (2) to create a small digital footprint for 21 CFR Part 11 (or Part 11 herein) validation (transaction begins at a point where participant clicks approval on the rendered PDF-consent document and ends with storage in the restricted access sFTP system).

#### Feature 8: Part 11 certification for eConsent

21 CFR Part 11 refers to the Code of Federal Regulations, established by the FDA to govern the criteria under which electronic records and signatures can be considered secure and trustworthy (i.e., equivalent in fidelity to paper records) [29]. Software cannot be Part 11 compliant alone – procedural controls are also required. VUMC has verified and documented its software and required procedural controls with the validation of the REDCap eConsent framework module. Validation of the VUMC eConsent framework was conducted by JAF Consulting, Inc. (Philadelphia, PA). The methods and procedural controls for establishing a Part 11 compliant eConsent framework have been documented and shared with REDCap Consortium partners interested in obtaining official certification. Those sites seeking to obtain certification have the option of utilizing the VUMC shared scripts, controls and validation SOPs, or hiring an independent Part 11 consultant to complete the validation process.

### Community-Engaged Technology Development

CESs and CIs were used to solicit community feedback during the development of our eConsent framework and feature set. CES allowed our research team to obtain feedback from stakeholder groups who were not familiar with eConsent or the goals of STRIDE. Specific groups engaged were study coordinators, regulatory teams, and community members/patients from underrepresented minority groups. In contrast, the CIs are embedded within each STRIDE site study team at the operations level and are not research or project “naïve” as is the case with CES participants. African American and Latino community representatives are integral team members who have drawn upon the wisdom of their respective communities to inform, at the earliest stages of the project, eConsent feature development, pilot testing, implementation, and dissemination. Community representatives were identified through prior participation in CESs and engagement with community-based organizations and local advocacy work. Vanderbilt community representatives are actively engaged in prison reform initiatives, community development boards, and financial coaching in underserved populations.

## Results

### Community Engagement Studios

We conducted nine CES to solicit feedback from community members and research assistants before and during the building and implementation of the eConsent framework, video library, and associated features. Table 1 summarizes the participant characteristics, focus, and recommendations of the Studios conducted at each of the three STRIDE sites.

**Table 1.** STRIDE community engagement studios – key recommendations

Location	Demographics	Topic(s)	Key recommendations
Nashville, TN	African American	Videos	Use animation for invasive procedures Videos should be short, informative, but clear and concise
Nashville, TN	Latino/Hispanic	Videos	Show diversity of ages and race/ethnicity Use videos to tell a story (e.g., greeting by nurse, inserting IV) Include subtitles for Spanish-speaking audience Use high-quality filmmaking
Worcester, MA	African American/Hispanic Community Leaders	Avatar	Avoid voices which sound computer generated Avoid 3D animation and cartoon-like 2D animation Avatars should appear as medical staff (e.g., lab coats) Avatar should not replace interaction with study staff Avatar helpful if consent sent out in advance
Birmingham, AL	Diverse group of individuals who previously participated in a clinical trial	Clinical trial participation	Train study staff to be patient, supportive, and knowledgeable Use clear and concise language in consent documents Partner with local organizations Increase diversity of study staff
Birmingham, AL	Diverse group of individuals who chose not to participate in a clinical trial	Clinical trial participation	Focus on value of research to the individual and future generations Address fears and lack of trust openly by incorporating testimonials
`Nashville, TN	Study recruiters/coordinators who utilize eConsent	eConsent framework	Provide option of paper consent to participants Consider breaking up sections by “pages” Few major differences between eConsent and paper consent
Nashville, TN	African American (many of whom attended the previous Studio)	eConsent framework/videos	Videos were representative, appropriate, and informative Provide both paper and eConsent options Useful if potentially can review in advance of consenting
Worcester, MA	Study recruiters/coordinators	Simulation training	Include additional training components including use of social media, building empathy, and addressing data privacy issues Offer tiers of training based on recruiter experience Develop interactive online training
Nashville, TN	Spanish speaking Latino/Hispanic adults	Clinical trial participation/eConsent platform	eConsent should not replace interaction with study staff Provide option of paper consent to participants Offer choice regarding eConsent add-ons (e.g., videos should be optional) Consider utilizing features to accommodate Spanish dialects (e.g., hover over to show differing translations for a word)

After reviewing sample videos, Studio attendees emphasized the importance of representing minority populations and encouraged creating vignettes by depicting supportive and helpful interactions with medical staff (e.g., greeting patients) and expressed a preference for use of animation to depict invasive procedures (e.g., lumbar puncture). Studio attendees noted that avatars should not replace interactions with study staff. In separate Studios, community members and study recruiters reviewed the completed eConsent framework. Recruiters recommended providing paper consent as an option for participants who may be uncomfortable with or who have difficulty accessing technology. Community members echoed the importance of offering a paper option and suggested sending the consent in advance of a meeting with the study coordinator. With a few individual preferences, the feedback from the community members regarding elements of eConsent and the video library was notably consistent across gender, age (19–84 years), and educational background (high school to post-graduate degree).

### Community Investigators

The STRIDE team has engaged seven CIs. The CIs provided feedback on the consent experience and individual elements of the consent including videos, avatars, and consent language. Their involvement informed the tailoring of features and documents to meet the varying levels of health literacy for prospective research participants. The input of the CIs helped to develop many of our eConsent features (e.g., avatars and actors/interactions in the videos) such that the depictions of people are more representative of the diversity that researchers hope to achieve within research studies.

### Video Library

Community feedback was used by our team to develop storyboards and voiceover scripts for eight procedures and/or research themes as topics for 10 videos that we created for the library (Table 2). The videos are short (1–4 min in length) and represent specific procedures with a voiceover explanation to describe each step of the process. In an effort to improve the accessibility of the videos to those of varying health literacy, the scripts were reviewed by the VUMC Effective Health Communication Core to reduce word count/sentence count and words per sentence. Scripts were generally written at a Flesch-Kincaid Grade Level of 6; however, the need to define acronyms involving medical terms such as magnetic resonance imaging and computed tomography resulted in final scripts at a 7–8th grade reading level.

**Table 2.** Common research procedures and informed consent elements in the eConsent video library

Biopsy (English and Spanish versions)	Genetic test
Blood sample	Lumbar puncture (LP)**
Computed tomography (CT) scan (with/without contrast)	Mammogram
Data privacy	Magnetic resonance imaging (MRI)
Echocardiogram**	Positron emission tomography (PET) scan

** Denotes videos developed in collaboration with the Recruitment Innovation Center (RIC).

### Implementation of eConsent

We are implementing and evaluating eConsent interventions as part of STRIDE studies and will report eConsent metrics and feedback in the context of individual study manuscripts. The eConsent feature set and framework were released to the global REDCap Consortium in March 2018. As of March 2020, >200 institutions have enabled the eConsent framework with approximately 140,000 eConsent transactions [11].

## Discussion

### General Workflow for REDCap-Based eConsent

**Fig. 2.**
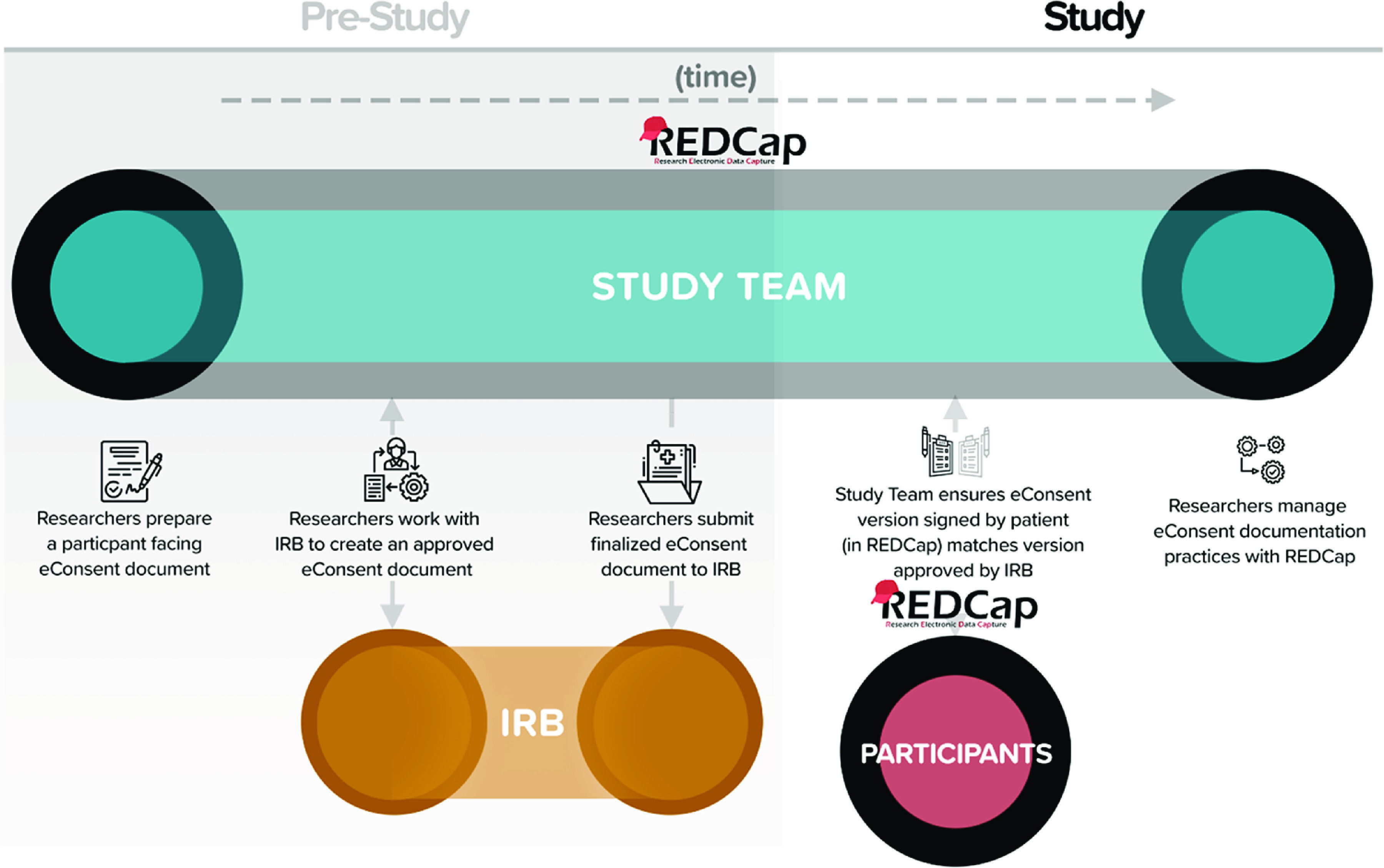
Workflow for eConsent development, IRB approval, and document management.

Based on pilot testing at other sites, we created a generalizable model for development and review of eConsent: (1) investigative team prepares a participant-facing eConsent survey instrument in REDCap and submits the document for IRB review (Fig. 2); (2) submission should include a static copy of the REDCap survey (an auto-generated, nondynamic PDF), links, transcripts for any embedded multimedia (e.g., videos), and a link to the REDCap eConsent survey for IRB review and approval, and (3) commitment from investigators to follow traditional procedures for consent documentation best practices and accountability. Investigators are responsible for ensuring that the version signed by participants matches the version approved by the IRB at the time of signature. Investigators must maintain version control of eConsents should they need to update/amend the consent document during the study. The REDCap eConsent framework automatically saves all consent documents to a central location so that researchers can easily sort, find, and review previously signed consent documents based on consent version, individual participant, and date.

Ethical considerations were identified during piloting that relate to the use of the analytics module which captures data on how a user interacts with the platform. Specifically, community members and IRB analysts voiced concerns about a participant’s right to know that their eConsent behavior is being recorded. To address these concerns, we developed standardized language to be included at the beginning of any eConsent in which this module will be enabled, “We would like to improve the consent experience for our research volunteers. Today, we will be collecting information about how you have used this electronic consent form”. This language was included as part of all STRIDE studies, vetted with community members who were familiar with the project and the specific analytics module, and the STRIDE site IRBs.

### STRIDE Pilot Testing

Refinements were made to the eConsent framework and workflow based on pilot testing and STRIDE team brainstorming. Refinements included:

1. Reviewer mode – a REDCap survey can be enabled for reviewer mode so that a member of the study team can summarize the eConsent document with a prospective participant without the need to complete required fields or having avatars enabled. This mirrors the workflow using a paper consent whereby a study team member provides a summary of the consent document to the participant before allowing them time to review.
2. Analytics dashboard – a dashboard was created to help visualize data captured by the analytics external module. The dashboard provides participant-level data detailing time spent on each page of the consent, cumulative time of the consent process, and time spent engaged with videos, avatars, or in-line descriptive pop ups.
3. eConsent preview – UAB piloted eConsent “previewing” in which a survey link is sent to a participant in advance of being officially consented in the clinic. In the preview mode, the wet signature field is removed allowing participants to read through the document, view videos, and interact with the avatars without signing.

### Lessons Learned and Challenges of the eConsent Framework

Feedback from the STRIDE sites has helped identify challenges related to use of eConsent in the real world and initiation of a series of best practices. Users are encouraged to engage their local REDCap teams early in their utilization of the eConsent framework to define project needs and timelines for implementation of specific features. To address regulatory-based challenges include balancing the flexibility needed by study teams with the security and limitations of the system imposed by the document vault and Part 11 compliance. Effectively, once a participant signs, confirms, and submits their eConsent, the record is “locked” in the virtual document vault and further changes are not permitted to the document. This design, intended to maintain the fidelity of the record, has posed some challenges to workflow, as “common sense” edits, cannot be saved/overwritten in the vault. Examples include going back into a record to correct a typographical error without having to initiate a new consent. We continue to develop REDCap infrastructure to mitigate this issue with the goal of making it easy to “do the right thing” for investigative teams, without creating unnecessary burden.

### Future Directions and Recommendations for Implementation of a Similar System

#### Part 11 compliance

As part of the STRIDE project, UAB and UMass are working with the VUMC REDCap team to update their local REDCap instances, implement the new features, and pursue Part 11 compliance certification for their eConsent frameworks.

#### Remote consenting and telemedicine

There is increased interest in the use of telemedicine, and by extension, remote consenting to help reach prospective patients/participants in rural healthcare settings, as well as to help address issues of health disparities, as rural communities are often underrepresented in research. Current evidence suggests that telemedicine, which can include video conferencing, web-based materials, and other media, under certain circumstances, can provide care equal to that of in-person interactions, with potentially lower cost [30,31]. Additionally, early adopters of remote consent via multimedia platforms have found that utilization of such platforms can streamline the consent process, are appealing to prospective participants as users are more likely to opt for the remote option of consent (if available), and users have demonstrated equal satisfaction with in person vs remote consent options [32,33].

Toward this end, some STRIDE exemplar studies utilized eConsent with a remote consenting component. For example, the UAB STRIDE exemplar study will send the eConsent for review, but not signature, by the participant prior to consenting in person at a clinic. Early results using remote eConsent with the Vanderbilt STRIDE exemplar study suggest that utilization of eConsent may increase the participation of minority pregnant women in prospective research. Researchers concluded that eConsent was effective for consenting women with low health literacy [34]. Further work over the course of the STRIDE project and more detailed data capture of participant interactions with the eConsent framework will assess the impact of the framework on recruitment and retention of minority participants in research studies and help to inform best practices for utilizing the framework in remote consenting.

#### Continuing the consent conversation

Consent is not just a one-time event but rather a continual process throughout the study. The eConsent framework provides a mechanism by which study teams can continue the consent conversation. Study teams may consider sending their eConsent public survey link in preview mode (i.e., no signature block but contains all other features) along with an appointment reminder and utilize this as a mechanism to revisit the consent and keep the conversation going with their research participants. This may help improve the accessibility of the study material as literature suggests that the informed consent conversation often uses easier words and is shorter than the consent document [35].

#### Managing eConsent for multicenter trials and sIRB

VUMC is working with the TIN on several multicenter studies to begin piloting processes and methods around using eConsent with multicenter studies and single IRB (sIRB) review [36]. The two main areas of focus have been regulatory (IRB review process) and consent management/housing of consents:

- Regulatory – sIRB review integrates seamlessly with the use of eConsent as multicenter studies often utilize a 2-part consent in which Part 1 represents study-specific information that is universal across all sites (reviewed by the IRB of record), and Part 2 is site specific (reviewed by the respective relying IRBs along with other relevant local context). The documentation described above (see section STRIDE Pilot Testing) provides a starting point for review by both the IRB of record and relying sites.
- Consent management – There are two models by which eConsents can be managed for multicenter studies: (1) the Data Coordinating Center (DCC) creates and manages a single eConsent repository in REDCap where individual site personnel may access information for only their sites or (2) each site maintains, builds, and stores their own eConsent documents locally and shares data directly with the DCC. While the setup for the DCC is greater if it manages and stores (on a local server) all of the eConsents for the study, the advantage is that version control and document management becomes streamlined, and for Part 11 applicable studies, only the site managing the consents needs to have a Part 11 validated instance of REDCap.

#### Video library

As VUMC (and other sites) continues to develop educational videos, we recognize an opportunity to develop a video library functionality within REDCap to make videos easily “discoverable” for users. Current work is seeking to embed a video library within REDCap that includes educational videos reviewed and approved by the VUMC IRB, with potential enhancements to allow other local IRBs to review and indicate their approvals. Ideally, users would be able to filter the video library based on procedure/theme and determine which institutions have registered their approval of a specific video. While we acknowledge that approval by one IRB will not ensure approval of videos by other IRBs and that videos must still be reviewed on a study by study basis with appropriate context, the video library may help facilitate eConsent review and approval in the current era of sIRBs.

#### eConsent training

The collaborative nature of REDCap users lends itself to rapid growth in this space as developers and researchers continue to test the framework with new use cases and advance methodologies to meet the needs of their stakeholders [37]. Despite the strengths of the REDCap platform, implementation and utilization of various features are only as good as the training and support available for these tools. There are a variety of resources to support building and designing eConsent projects in REDCap including tutorials that detail step-by-step project build-out, webinars to describe new eConsent features, and community involvement in the development process, as well as guidance available through the TIN to support studies seeking to use eConsent [36,38–40].

## Conclusion

The eConsent features described herein are being circulated within the REDCap Consortium and disseminated using webinars and “Toolbox” sharing mechanisms via STRIDE online materials and the TIN Recruitment Innovation Center [12,14]. We continue to refine features based on continued community-engaged technology development methodologies. We are combining the STRIDE project experience with anecdotal feedback from our larger REDCap worldwide research community that adopted the eConsent framework to define and prioritize new “wish-list” features for development. These include embedding a video library within REDCap, improving avatar functionality for voiceovers (including non-English languages), and possibly integrating eConsent with electronic health record systems. We look forward to using the implementation of eConsent projects to gather evidence to support our hypothesis for STRIDE that the eConsent framework provides an opportunity to improve access of underrepresented minority groups for translational research by facilitating engagement with the consent process.

## References

[1] Lorell BH , et al. Informed consent in clinical research: Consensus recommendations for reform identified by an expert interview panel. Clinical Trials (London, England) 2015; 12(6): 692–695. 10.1177/1740774515594362 PMC4657389 26178662

[2] Shenoy P . Electronic informed consenting: A boon to modernize consenting process. Perspectives in Clinical Research. 2015; 6(4): 173–174. 26623385 10.4103/2229-3485.167091 PMC4640006

[3] Rothwell E , et al. A randomized controlled trial of an electronic informed consent process. Journal of Empirical Research on Human Research Ethics (JERHRE) 2014; 9(5): 1–7. 10.1177/1556264614552627 PMC5847281 25747685

[4] *Use of Electronic Informed Consent in Clinical Investigations Questions and Answers Guidance for Industry Draft Guidance* [Internet]. United States Food and Drug Administration (FDA), 2015. (https://www.fda.gov/downloads/Drugs/GuidanceComplianceRegulatoryInformation/Guidances/UCM436811.pdf?source=govdelivery&utm_medium=email&utm_source=govdelivery)

[5] Flory J , Emanuel E. Interventions to improve research participants’ understanding in informed consent for research: a systematic review. Journal of the American Medical Association. 2004; 292(13): 1593–1601. 15467062 10.1001/jama.292.13.1593

[6] Tait AR , et al. Patient comprehension of an interactive, computer-based information program for cardiac catheterization: A comparison with standard information. Archives of Internal Medicine 2009; 169(20): 1907–1914. 19901144 10.1001/archinternmed.2009.390 PMC2776761

[7] Warriner AH , et al. A pragmatic randomized trial comparing tablet computer informed consent to traditional paper-based methods for an osteoporosis study. Contemporary Clinical Trials Communications 2016; 3: 32–38. 29736454 10.1016/j.conctc.2016.02.003 PMC5935867

[8] Frelich M , Bosler M , Gould JC . Research Electronic Data Capture (REDCap) electronic Informed Consent Form (eICF) is compliant and feasible in a clinical research setting. International Journal of Clinical Trials 2015; 2(3): 51–55.

[9] *Seeing Beyond the Margins: Challenges to Informed Inclusion of Vulnerable Populations in Research - Sarah Gehlert, Jessica Mozersky, 2018* [Internet] [cited 2019 Sep 6]. (https://journals.sagepub.com/doi/full/10.1177/1073110518766006) 10.1177/1073110518766006 PMC6077979 30093794

[10] *Use of Electronic Informed Consent in Clinical Investigations Questions and Answers Guidance for Industry Draft Guidance* [Internet]. United States Food and Drug Administration (FDA), 2015. (https://www.fda.gov/downloads/Drugs/GuidanceComplianceRegulatoryInformation/Guidances/UCM436811.pdf?source=govdelivery&utm_medium=email&utm_source=govdelivery)

[11] Harris PA , et al. Research electronic data capture (REDCap) – a metadata-driven methodology and workflow process for providing translational research informatics support. Journal of Biomedical Informatics 2009; 42(2): 377–381. 18929686 10.1016/j.jbi.2008.08.010 PMC2700030

[12] *The REDCap consortium: Building an international community of software platform partners. - PubMed - NCBI* [Internet] [cited 2019 Sep 6]. (https://www.ncbi.nlm.nih.gov/pubmed/?term=The+REDCap+consortium%3A+Building+an+international+community+of+software+platform+partners) 10.1016/j.jbi.2019.103208 PMC7254481 31078660

[13] Zerhouni EA Translational and clinical science – time for a new vision. The New England Journal of Medicine 2005; 353(15): 1621–1623. 16221788 10.1056/NEJMsb053723

[14] Bernard GR , et al. A collaborative, academic approach to optimizing the national clinical research infrastructure: The first year of the Trial Innovation Network. Journal of Clinical and Translational Science 2018; 2(4): 187–192. 31011433 10.1017/cts.2018.319 PMC6474372

[15] *DatStat eConsent* [Internet] DatStat. 2017 [cited 2019 Jan 11]. (https://www.datstat.com/datstat-econsent)

[16] *Mytrus, Inc.* [Internet] [cited 2019 Jan 11]. (https://www.mytrus.com/en/products-services/enroll)

[17] Hallinan ZP , et al. Barriers to change in the informed consent process: A systematic literature review. IRB 2016; 38(3): 1–10. 27301167

[18] Ford JG , et al. Barriers to recruiting underrepresented populations to cancer clinical trials: A systematic review. Cancer 2008; 112(2): 228–242. 18008363 10.1002/cncr.23157

[19] Baker DW. PERSPECTIVE: The meaning and the measure of health literacy. Journal of General Internal Medicine N Y. 2006; 21(8): 878–883. 10.1111/j.1525-1497.2006.00540.x PMC1831571 16881951

[20] National Action Plan to Improve Health Literacy | Healthy People 2020 [Internet] [cited 2020 Apr 14]. (https://www.healthypeople.gov/2020/tools-resources/evidence-based-resource/national-action-plan-improve-health-literacy)

[21] *Culturally appropriate storytelling to improve blood pressure: A randomized trial* – PubMed – NCBI [Internet] [cited 2019 Sep 6]. (https://www.ncbi.nlm.nih.gov/pubmed/21242364)

[22] *An Innovative Simulation-based Community-engaged Intervention for Training Research Assistants in Culturally Appropriate Informed Consent. – PubMed - NCBI* [Internet] [cited 2019 Sep 6]. (https://www.ncbi.nlm.nih.gov/pubmed/30581168)

[23] George S , Duran N , Norris K. A systematic review of barriers and facilitators to minority research participation among African Americans, Latinos, Asian Americans, and Pacific Islanders. American Journal of Public Health 2013; 104(2): e16–e31. 24328648 10.2105/AJPH.2013.301706 PMC3935672

[24] Powell LR , et al. Psychometric development of the research and knowledge scale. Medical Care 2017; 55(2): 117–124. 27579914 10.1097/MLR.0000000000000629 PMC5233643

[25] Andrulis DP , Brach C. Integrating literacy, culture, and language to improve health care quality for diverse populations. American Journal of Health Behavior 2007; 31(Suppl 1): S122–S133 17931131 10.5555/ajhb.2007.31.supp.S122 PMC5091931

[26] *Development work for REDCap External Modules/Packages to support a standardized Hook/Plugin framework and management mechanism: vanderbilt/redcap-external-modules* [Internet]. Vanderbilt Institute for Clinical and Translational Research, 2019 [cited 2019 Jan 11]. (https://github.com/vanderbilt/redcap-external-modules)

[27] Joosten YA , et al. Community engagement studios: A structured approach to obtaining meaningful input from stakeholders to inform research. Academic Medicine: Journal of the Association of American Medical 2015; 90(12): 1646–1650. 10.1097/ACM.0000000000000794 PMC4654264 26107879

[28] *Oddcast - Technology* [Internet] [cited 2019 Jan 11]. (http://www.oddcast.com/tech/)

[29] *eCFR — Code of Federal Regulations* [Internet] [cited 2018 Dec 28]. (https://www.ecfr.gov/cgi-bin/text-idx?SID=3ee286332416f26a91d9e6d786a604ab&mc=true&tpl=/ecfrbrowse/Title21/21tab_02.tpl)

[30] McConnochie KM Webside manner: A key to high-quality primary care telemedicine for all. Telemedicine and e-Health Off American Telemedicine Association 2019; 25(11), 1007–1011 10.1089/tmj.2018.0274 30648924

[31] *Telehealth helps Einstein Medical Center slash readmissions, boost value-based care* [Internet]. Healthcare IT News. 2018 [cited 2019 Jan 22]. (https://www.healthcareitnews.com/news/telehealth-helps-einstein-medical-center-slash-readmissions-boost-value-based-care)

[32] Schallhorn SC , et al. Informed consent in refractive surgery: In-person vs telemedicine approach. Clinical ophthalmology (Auckland, N.Z.) 2018; 12: 2459–2470. 10.2147/OPTH.S183249 PMC6278698 30568424

[33] Haussen DC , et al. Utilization of a smartphone platform for electronic informed consent in acute stroke trials. Stroke 2017; 48(11): 3156–3160. 28986425 10.1161/STROKEAHA.117.018380

[34] Phillippi JC , et al. Electronic informed consent to facilitate recruitment of pregnant women into research. Journal of Obstetric, Gynecologic, & Neonatal Nursing. 2018; 47(4): 529–534. 10.1016/j.jogn.2018.04.134 PMC6071311 29758172

[35] Koyfman SA , et al. Informed consent conversations and documents: A quantitative comparison. Cancer 2016; 122(3): 464–469. 26505269 10.1002/cncr.29759 PMC4724216

[36] *Trial Innovation Network* [Internet] [cited 2019 Jan 22]. (https://trialinnovationnetwork.org/)

[37] Harris PA , et al. REDCap Consortium. The REDCap consortium: Building an international community of software platform partners. Journal of Biomedical Informatics. 2019; 95: 103208. 31078660 10.1016/j.jbi.2019.103208 PMC7254481

[38] Harris PA. *REDCap - New Modules Supporting eConsent + EHR workflow and data interoperability* [Internet] [cited 2019 Jan 11]. (https://trialinnovationnetwork.org/wp-content/uploads/2018/09/2018-09-19-11.00-REDCap-New-Modules-Supporting-eConsent-_-EHR-workflow-and-data-interoperability.mp4)

[39] Lawrence C. *STRIDE & eConsent: Moving Towards Personalized Informed Consent* [Internet] [cited 2019 Jan 11]. (https://trialinnovationnetwork.org/wp-content/uploads/2017/12/2017-12-14-11.00-STRIDE-_-eConsent_-Moving-Towards-Personalized-Informed-Consent.mp4)

[40] *ITHS | Using REDCap to consent research participants* [Internet] [cited 2019 Jan 22]. (https://www.iths.org/blog/news/redcap-tip/using-redcap-to-consent-research-participants/)

